# Supplementary material for: Metagenomic and satellite analyses of red snow in the Russian Arctic
Source: PeerJ. 2015 Dec 10;3:e1491. doi: 10.7717/peerj.1491 (PMC4690372; doi:10.7717/peerj.1491)
Supplement: Supplemental Information 1 [file peerj-03-1491-s001.docx]

**Metagenomic and Satellite Analyses of Red Snow in the Russian Arctic**

Nao Hisakawa, Steven Quistad, Eric Hester, Daria Martynova, Heather Maughan, Enric Sala, Maria Gavrilo, Forest Rohwer

**Supplementary Materials**

**Methods**

Satellite image analysis

Satellite images were obtained for the following locations and dates: Nansen Island in Franz Josef Land, Russia, in July of 1986, 2002, and 2006; Harding Ice Field, USA, in August of 2000, 2005, 2010, and 2014; Olympic National Park, USA, in July of 1996, 2002, 2008, and 2013; Rocky Mountains, Canada, in July of 1996, 2003, 2009, and 2014; Sierra Nevada, USA, in July of 1999, 2003, 2009, and 2013; Glacier National Park, USA, in July of 2002, 2006, 2010, and 2013; Mittivakkat Glacier, Greenland, in August of 1990, 2001, and 2014; Svalbard Archipelago, Norway, in July of 1989, 2002, 2006, and 2014; Grossglockner Mountain, Austria, in July of 1987, 2002, 2009, and August of 2013; Himalayas, India, in August of 1999, 2002, 2009, and 2014; and Mt Cook, New Zealand, in December of 1990, 2002, 2008, and 2014.

For each location, images acquired over multiple years were used to study red snow coverage over time. Images acquired in July and August for each year studied were used for the analysis in Northern Hemisphere locations because red snow becomes visible in July (Lutz et al. 2014; Remias et al. 2005) and can bloom on the snow surface into August (Takeuchi 2009). Red snow is not as visible in the snow-fields during May and June (Remias et al. 2005; Takeuchi 2009), so images from those months were not used. Images taken in July were chosen over images taken in August because red snow first blooms in July. For Mt. Cook, New Zealand, images acquired in December were used, as that is the beginning of the summer in the Southern Hemisphere. Not all of the available images acquired in the Landsat Archive database were suitable for analysis. Images acquired in the dark or images with extensive cloud cover that obstructed the view of the land surface were excluded from the analysis because they posed challenges in detecting surface snow color. The remaining images were visually inspected and sets of images that had no cloud cover over the area of interest were chosen for the analysis. Red snow abundances in Franz Josef Land over three different years were compared, including images from 1986 (taken on July 20 by Landsat 5), 2002 (taken on July 17 by Landsat 7), and 2006 (taken on July 2, 2006 by Landsat 5). The same red and green band designations (described in main article Methods) were used for images from both satellites Landsats 5 and 7.

A list of images that were used is given in the Supplementary file titled “Landsat IDs”.

**References:**

Lutz S, Anesio AM, Villar SEJ, and Benning LG. 2014. Variations of algal communities cause darkening of a Greenland glacier. *Fems Microbiology Ecology* 89:402-414. Doi 10.1111/1574-6941.12351

Remias D, Lutz-Meindl U, and Lutz C. 2005. Photosynthesis, pigments and ultrastructure of the alpine snow alga Chlamydomonas nivalis. *European Journal of Phycology* 40:259-268. Doi 10.1080/09670260500202148

Takeuchi N. 2009. Temporal and spatial variations in spectral reflectance and characteristics of surface dust on Gulkana Glacier, Alaska Range. *Journal of Glaciology* 55:701-709.
