# Supplementary figures and images for: Metagenomic and satellite analyses of red snow in the Russian Arctic
Source: PeerJ. 2015 Dec 10;3:e1491. doi: 10.7717/peerj.1491 (PMC4690372; doi:10.7717/peerj.1491)

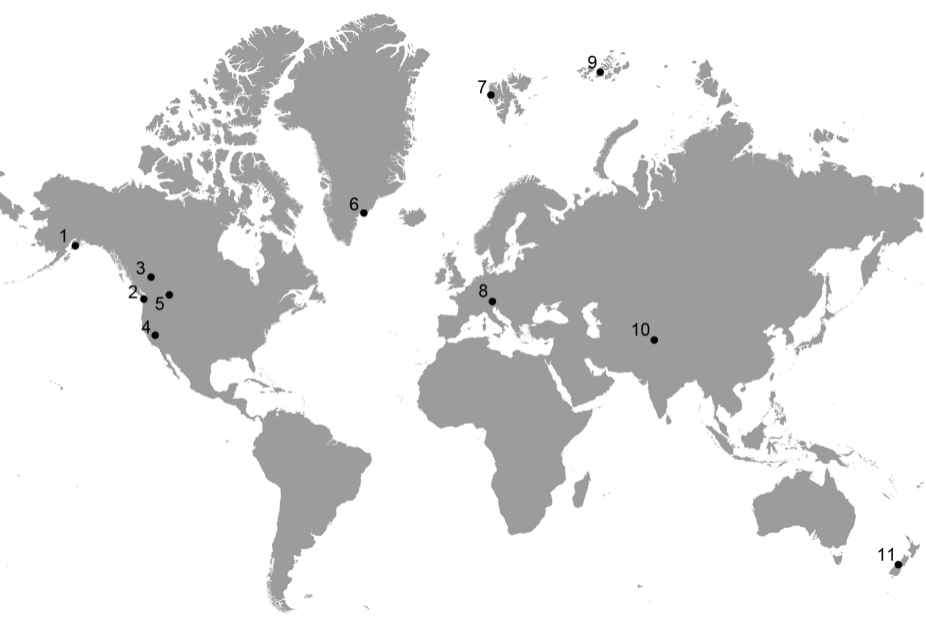

Supplement: Figure S1 — Black points indicate the locations analyzed for red snow content. Harding Ice Field, United States (1); Olympic National Park, United States (2); Rocky Mountains, Canada (3); Sierra Nevada Mountain Range, United States (4); Glacier National Park, United States (5); Mittavikkat Glacier, Greenland (6); Svalbard Archipelago, Norway (7); Grossglockner Mountain, Austria (8); Nansen Island, Franz Josef Land (9); Himalaya Mountain Range, India (10); Mt. Cook, New Zealand (11). [file peerj-03-1491-s002.pdf]

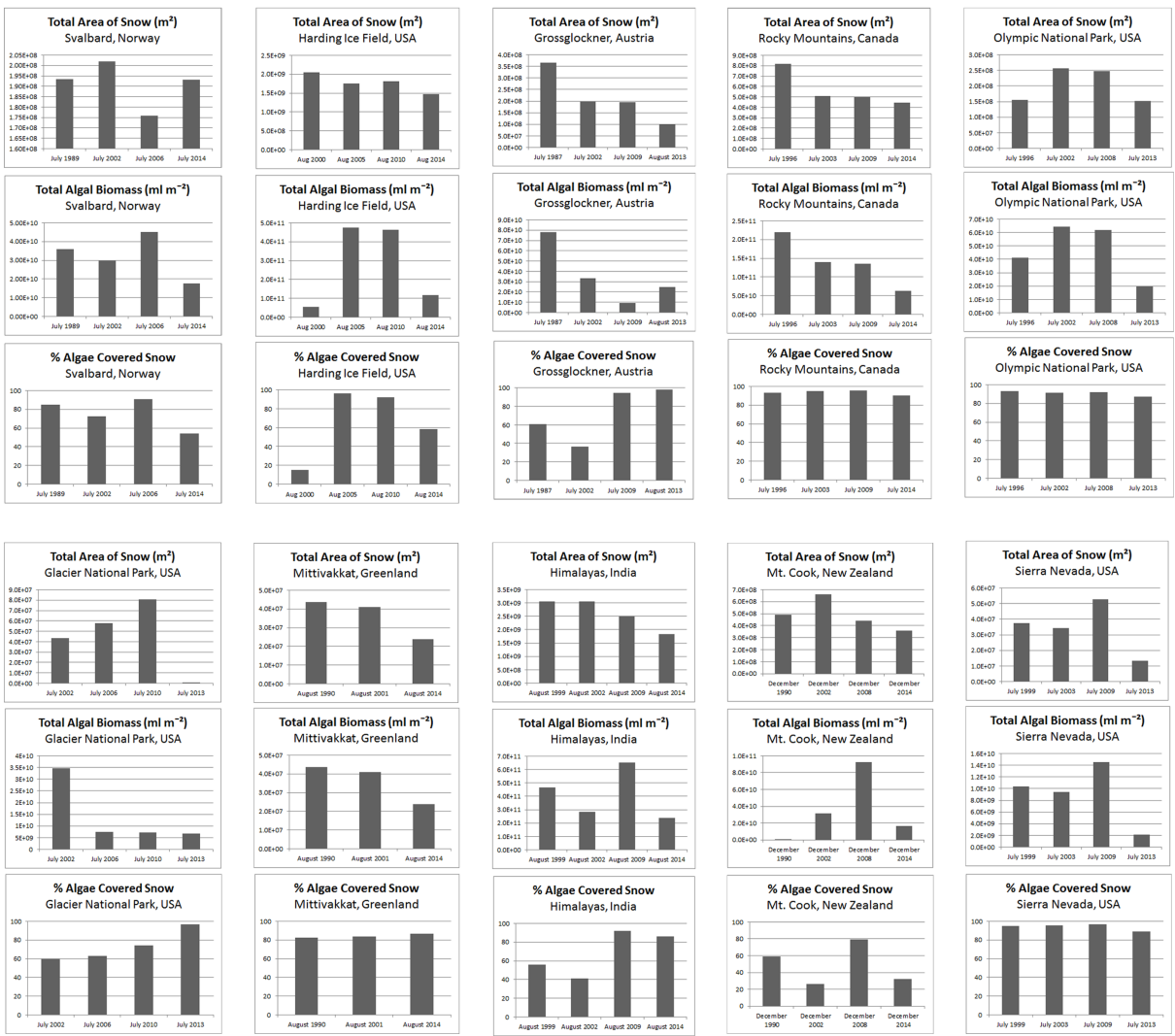

Supplement: Figure S2 — A time series comparison of the total area of snow and sea ice, total algal biomass, and percentage of total snow that is covered with algae at ten different study sites. [file peerj-03-1491-s003.pdf]

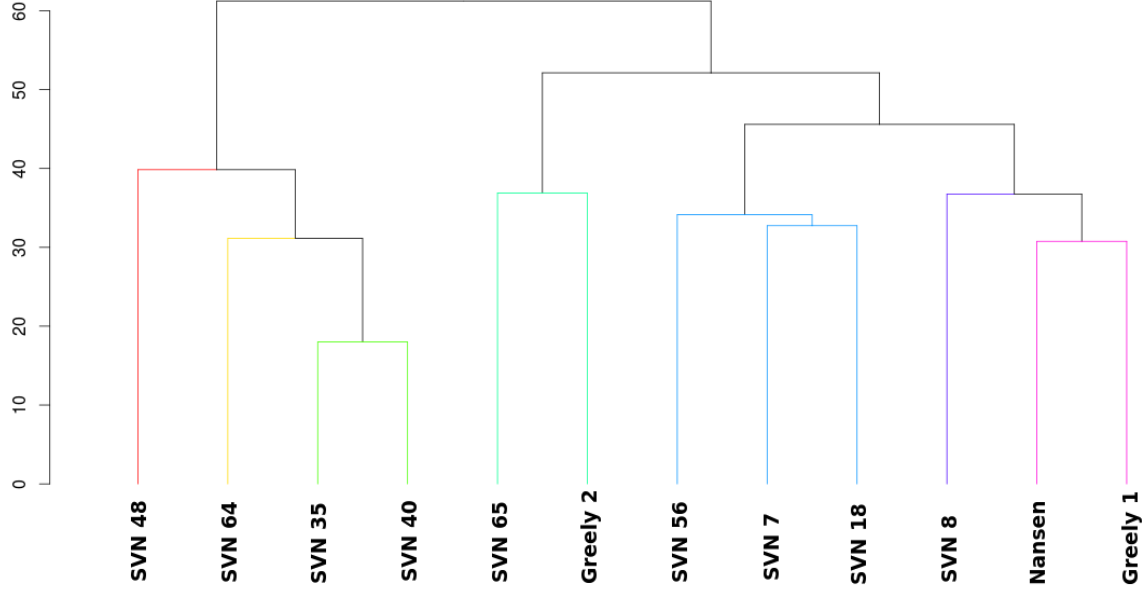

Supplement: Figure S3 — Clustering of snow and red snow communities based on similarities in taxonomic composition. Colored branches indicate significant clusters, with each color representing one cluster. Labels with ‘SVN’ correspond to white snow samples and ‘Greely 1’, ‘Greely 2’, and ‘Nansen’ correspond to red snow samples. [file peerj-03-1491-s004.pdf]

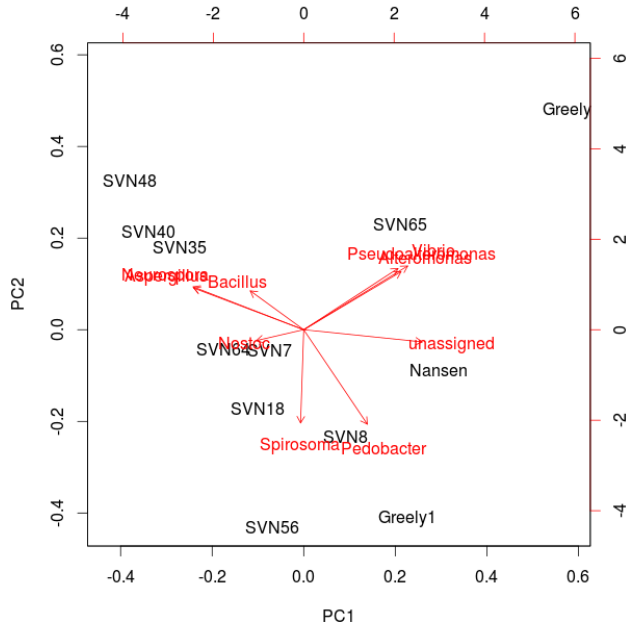

Supplement: Figure S4 — Plot from principal components analysis of red snow and snow community taxonomic composition. Arrows indicate over-representation of certain taxa (red text) in particular red snow or snow communities (black text). Labels are described in the legend for Fig. S3. [file peerj-03-1491-s005.pdf]

Proportion of reads assigned to Chlorophyta

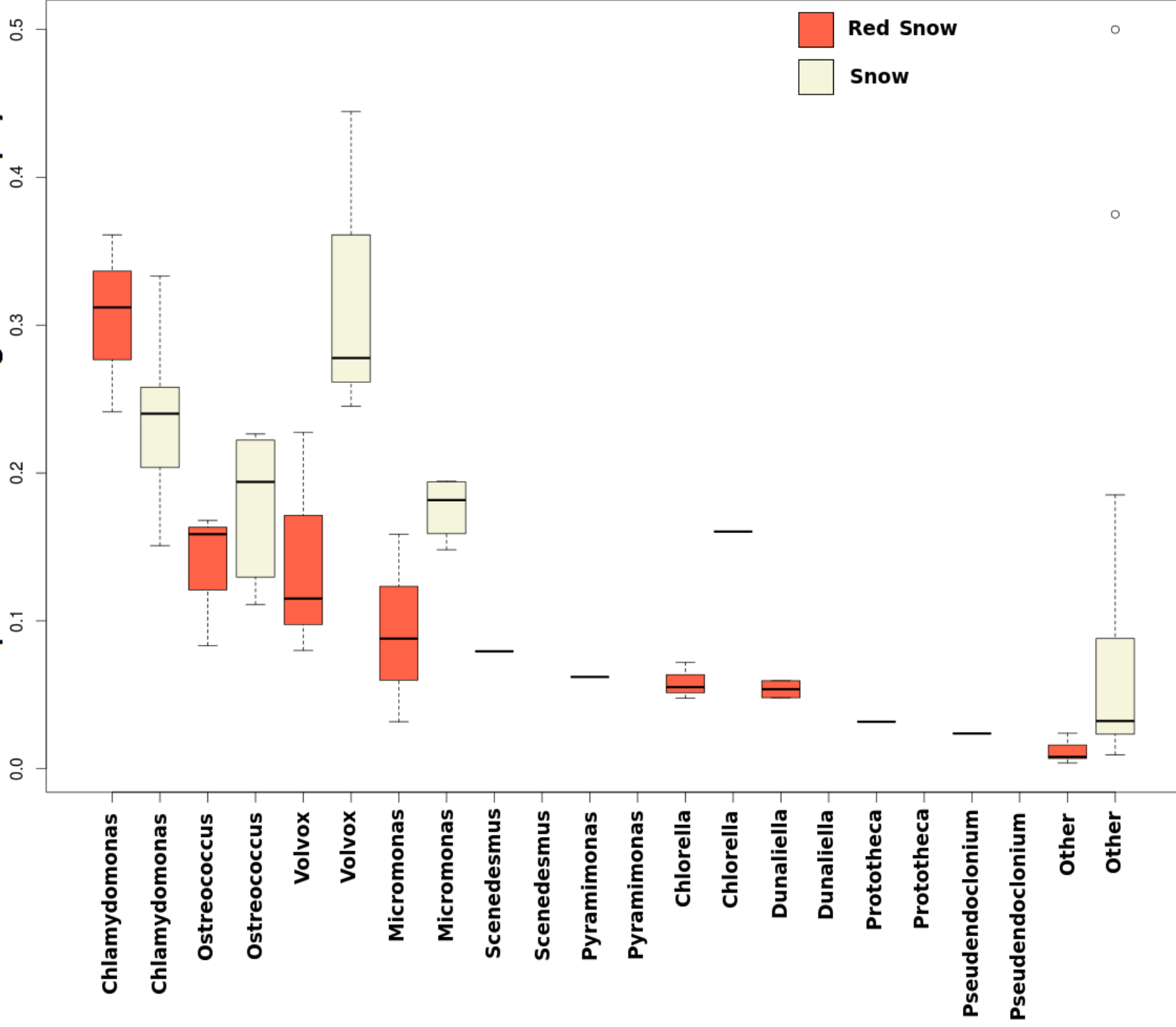

Supplement: Figure S5 — Bar plots showing genus level comparisons of reads assigned to Chlorophyta. The y-axis shows the proportion of reads assigned to the phylum Chlorophyta for each genus. Red bars indicate data from red snow samples and khaki bars indicate data from snow. Snow metagenomes did not contain any reads from the following genera: Scenedesmus, Pyramimonas, Dunaliella, Prototheca, and Pseudendoclonium. [file peerj-03-1491-s006.pdf]

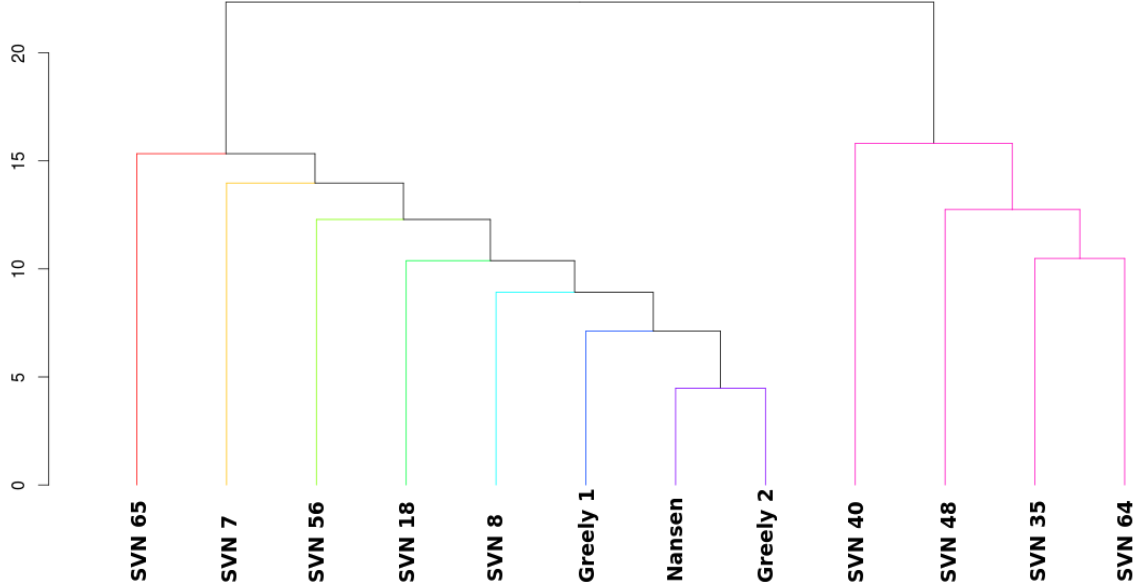

Supplement: Figure S6 — Clustering of snow and red snow samples based on similarities in functions encoded by each community sample. Colored branches indicate significant clusters, with each color representing one cluster. Labels are described in the legend for Fig. S3. [file peerj-03-1491-s007.pdf]

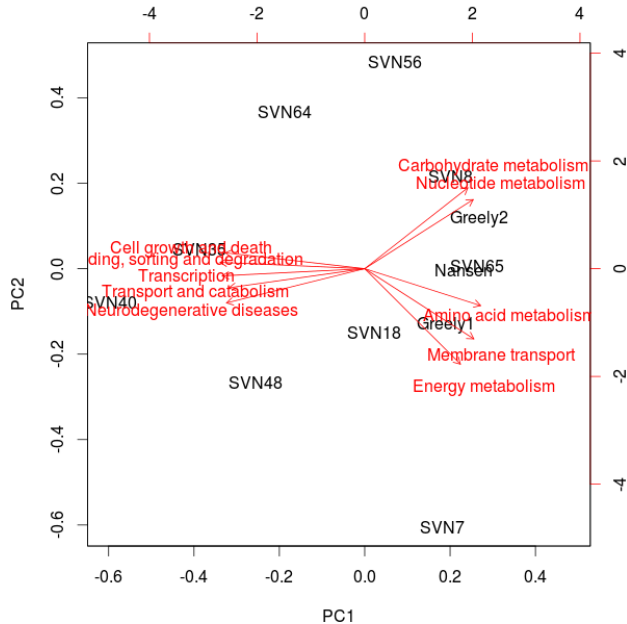

Supplement: Figure S7 — Principal components analysis of red snow and snow community functions. Arrows indicate over-representation of certain functional categories (red text) in particular red snow or snow communities (black text). Labels are described in the legend for Fig. S3. [file peerj-03-1491-s008.pdf]
