# Supplementary material for: Metagenomic and satellite analyses of red snow in the Russian Arctic
Source: PeerJ. 2015 Dec 10;3:e1491. doi: 10.7717/peerj.1491 (PMC4690372; doi:10.7717/peerj.1491)
Supplement: Table S1 — The relationship between reflectance band ratio values, C. nivalis biomass, and the proposed level of algae abundance, extrapolated from a positive linear correlation between reflectance band ratio and algal biomass that was shown in previous research (Takeuchi et al., 2006). [file peerj-03-1491-s009.docx]

Supplementary Table 1. The relationship between reflectance band ratio values, *C. nivalis* biomass, and the proposed level of algae abundance, extrapolated from a positive linear correlation between reflectance band ratio and algal biomass that was shown in previous research (Takeuchi et al. 2006).

| **Red to green reflectance band ratio** | **Algal biomass (ml mˉ²)** | **Mean algal biomass (ml mˉ²)** | **Algae abundance level** |
| --- | --- | --- | --- |
| < 1 | < 100 | Not applicable | Insignificant |
| 1 – 1.04 | 100 – 168.75 | 134.375 | Low |
| 1.04 – 1.08 | 168.75 – 237.5 | 203.125 | Medium |
| 1.08 – 1.12 | 237.5 – 300 | 268.75 | High |
| > 1.12 | > 300 | 300 | Very high |
