# Supplementary material for: Metagenomic and satellite analyses of red snow in the Russian Arctic
Source: PeerJ. 2015 Dec 10;3:e1491. doi: 10.7717/peerj.1491 (PMC4690372; doi:10.7717/peerj.1491)
Supplement: Table S2 [file peerj-03-1491-s010.docx]

Supplementary Table 2. Metagenomic samples.

| **Sample name** | **GPS** | **Date** | **Character of snow** | **MG-RAST ID** | **Reference** |
| --- | --- | --- | --- | --- | --- |
| SVN7 | 78.92, 11.94 | April 19 2008 | spring snowpack | 4451070.3 | (Maccario et al. 2014) |
| SVN8 | 78.92, 11.94 | April 19 2008 | spring snowpack | 4451073.3 | (Maccario et al. 2014) |
| SVN18 | 78.92, 11.94 | April 25 2008 | spring snowpack | 4451075.3 | (Maccario et al. 2014) |
| SVN35 | 78.92, 11.94 | May 9 2008 | spring snowpack | 4451065.3 | (Maccario et al. 2014) |
| SVN40 | 78.92, 11.94 | May 13 2008 | spring snowpack | 4451076.3 | (Maccario et al. 2014) |
| SVN48 | 78.92, 11.94 | May 20 2008 | spring snowpack | 4451068.3 | (Maccario et al. 2014) |
| SVN56 | 78.92, 11.94 | May 27 2008 | spring snowpack | 4451069.3 | (Maccario et al. 2014) |
| SVN64 | 78.92, 11.94 | June 2 2008 | spring snowpack | 4451072.3 | (Maccario et al. 2014) |
| SVN65 | 78.92, 11.94 | June 2 2008 | spring snowpack | 4451071.3 | (Maccario et al. 2014) |
| Greely 1 | 81.00, 58.30 | August 15 2013 | red | 4614610.3 | This paper |
| Greely 2 | 81.00, 58.30 | August 15 2013 | red | 4614611.3 | This paper |
| Nansen | 80.55, 54.11 | August 23 2013 | red | 4614609.3 | This paper |

Maccario L, Vogel TM, and Larose C. 2014. Potential drivers of microbial community structure and function in Arctic spring snow. *Frontiers in Microbiology* 5. ARTN 413 DOI 10.3389/fmicb.2014.00413
