# Supplementary material for: Metagenomic and satellite analyses of red snow in the Russian Arctic
Source: PeerJ. 2015 Dec 10;3:e1491. doi: 10.7717/peerj.1491 (PMC4690372; doi:10.7717/peerj.1491)
Supplement: Supplemental Information 2 — This file lists the Landsat IDs used for satellite image analysis of snow/ice/red snow abundances. Images can be acquired freely at: http://earthexplorer.usgs.gov/. [file peerj-03-1491-s011.docx]

Landsat Image ID# used for red snow paper

Franz Josef Land

LC82030012015186LGN00 (2015)

LT51970022006183KIS00 (2006)

LE71950022002198SGS00 (2002)

LT51960021986201KIS00 (1986)

Harding Ice Field, Alaska, USA

LC80680182014213LGN00 (2014) LT50690182010241GLC00 (2010)

LT50680182005220PAC00 (2005)

LE70690182000222AGS00 (2000)

Svalbard, Norway

LC80292402014196LGN00 (2014)

LT52160042006204KIS00 (2006)

LE72170032002192EDC00 (2002)

LT52170031989212KIS00 (1989)

Grossglockner, Austria

LC81920272013215LGN00 (2013)

LT51920272009204MOR00 (2009) LE71920272002209EDC00 (2002)

LT51920271987208AAA02 (1987)

Rocky Mountains, Canada

LC80470232014210LGN00 (2014)

LT50470232009212PAC02 (2009)

LT50470232003212PAC02 (2003)

LT50470231996209PAC00 (1996)

Olympic National Park, USA

LC80470272013191LGN00 (2013)

LT50470272008194PAC01 (2008) LT50470272002193LGS01 (2002)

LT50470271996193PAC04 (1996)

Glacier National Park, USA

LC80410262013197LGN00 (2013)

LT50410262010189EDC00 (2010)

LT50410262006178PAC01 (2006)

LE70410262002191EDC00 (2002)

Mittivakkat, Greenland

LC82310142014219LGN00 (2014)

LE72320142001214AGS00 (2001)

LT52310141990217KIS00 (1990)

Himalayas, India

LC81490352014205LGN00 (2014)

LC81490352014237LGN00 (2014)

LT51490352009223KHC00 (2009) LE71490352002228SGS00 (2002)

LT51490351999228AAA02 (1999)

Mt. Cook, New Zealand

LC80750902014358LGN00 (2014)

LT50750902008358HOA00 (2008)

LE70750902002365EDC00 (2002)

LT40750901990364XXX03 (1990)

Sierra Nevada, USA
LC80420342013188LGN00 (2013)

LT50420342009193PAC01 (2009)

LT50420342003193PAC03 (2003)

LT50420341999198XXX01 (1999)
